# Sublethal Pyrethroid Insecticide Exposure Carries Positive Fitness Effects Over Generations in a Pest Insect

**DOI:** 10.1038/s41598-019-47473-1

**Published:** 2019-08-05

**Authors:** Aigi Margus, Saija Piiroinen, Philipp Lehmann, Santtu Tikka, Juha Karvanen, Leena Lindström

**Affiliations:** 1 Centre of Excellence in Biological Interactions Research, Department of Biological and Environmental Science, University of Jyväskylä, PO Box 35, Jyväskylä, FI-40014 Finland; 2 Department of Zoology, Stockholm University, Svante Arrheniusväg 18b, SE-10691 Stockholm, Sweden; 3 Department of Mathematics and Statistics, University of Jyväskylä, Jyväskylä, PO Box 35, FI-40014 Finland

**Keywords:** Invasive species, Evolutionary ecology

## Abstract

Stress tolerance and adaptation to stress are known to facilitate species invasions. Many invasive species are also pests and insecticides are used to control them, which could shape their overall tolerance to stress. It is well-known that heavy insecticide usage leads to selection of resistant genotypes but less is known about potential effects of mild sublethal insecticide usage. We studied whether stressful, sublethal pyrethroid insecticide exposure has within-generational and/or maternal transgenerational effects on fitness-related traits in the Colorado potato beetle (*Leptinotarsa decemlineata*) and whether maternal insecticide exposure affects insecticide tolerance of offspring. Sublethal insecticide stress exposure had positive within-and transgenerational effects. Insecticide-stressed larvae had higher adult survival and higher adult body mass than those not exposed to stress. Furthermore, offspring whose mothers were exposed to insecticide stress had higher larval and pupal survival and were heavier as adults (only females) than those descending from control mothers. Maternal insecticide stress did not explain differences in lipid content of the offspring. To conclude, stressful insecticide exposure has positive transgenerational fitness effects in the offspring. Therefore, unsuccessful insecticide control of invasive pest species may lead to undesired side effects since survival and higher body mass are known to facilitate population growth and invasion success.

## Introduction

Invasive species pose a serious threat not only to agriculture, and the health of humans and animals, but also to biodiversity and ecosystem functioning^1,2^. Thus, it is important to understand the ecological and evolutionary factors that influence invasion success. Stress tolerance and adaptation to stressful environments are among the most important factors contributing to invasion success^3,4^. Stress can be defined as changes in the external or internal environment that threaten the maintenance of homoeostasis^5,6^. High stress-tolerance or organismal flexibility (e.g. behavioural or physiological) may contribute to invasion success by enabling invasive species to persist under unfavourable environmental conditions and allow time for adaptation to occur^3,4^. Stress may also be adaptive (via the process of genetic assimilation) by releasing phenotypic variation that contributes to fitness, or by facilitating developmental expression of beneficial traits that are phenotypically neutral under normal conditions^7,8^. Thus, in order to prevent invasions, it is important to understand how invasive species respond to stress and what the evolutionary consequences of stress are.

Many invasive species are insect pests and insecticides are commonly used to control them. However, insecticides do not only form a strong selection pressure but can also be a major stress factor when exposure is sublethal. Insect pests can be exposed to sublethal levels of insecticide in several ways; for example, as a result of an improper application or due to the degradation of the insecticide by abiotic factors, such as sunlight, rainfall or temperature^9^. Studies exploring sublethal insecticide exposures in insecticide resistance are rare but relevant due to their potential consequences also at the community level (i.e. community stress due to the changes in the species interactions)^10^. While insecticide stress generally has negative fitness effects^11^, it can at times be advantageous and increase fitness^12–14^. This phenomenon, where exposure to low levels of stress can induce stimulatory effects but is lethal at higher exposure levels, is known as hormesis^14^. This phenomenon has been demonstrated on maize weevil (*Sitophilus zeamais*) where exposure to sublethal doses of pyrethroid insecticide lead to a peak in the net reproductive rate^15^. Nevertheless, both positive and negative insecticide stress-induced modifications can have adaptive importance as these may be carried over to the next generation and persist across multiple generations^7,16,17^ through transgenerational effects.

Transgenerational effects occur when the phenotype of the offspring is influenced by the phenotype or environment of its parents^18–20^. Mousseau and Fox^18^ suggest that these transgenerational effects are most often seen between mother and offspring. This is because mothers can contribute to offspring development through a range of inputs via nutrition of the egg, transfer of immune factors or epigenetic mechanisms^18,21,22^. Transgenerational stress effects have received attention because of their significance from an evolutionary point of view^23^. However, to date, only a few studies have examined transgenerational stress in the context of insect pest invasions^13,16^. Considering the harm that invasive pest species pose to the environment and agriculture, it is important to study how insecticide stress affects performance and population dynamics within a generation, and whether sublethal doses lead to transgenerational cost or benefits.

The Colorado potato beetle (*Leptinotarsa decemlineata* Say.) is a notorious pest of potato (*Solanum tuberosum*). The beetle is native to Mexico and the south-western parts of the United States but can nowadays be found from the sub-tropical to temperate northern hemisphere^24,25^, and it is predicted to continue to expand its range rapidly^26,27^. The control of the beetle is heavily based on insecticides^28,29^. It is an excellent species to study insecticide stress within- and across generations in the context of invasive species. Due to its complex life-history combined with high selection pressure and rapid adaptation, the beetle has developed resistance to most classes of insecticides^28,30^. This means, that for already resistant populations, an insecticide application is likely to cause stress instead of lethal effects.

In the present study we investigated within- and transgenerational (maternal) effects of sublethal insecticide stress on several fitness-associated traits; survival, development time, body mass and lipid content. By rearing beetles for two generations we could investigate within-generational responses to insecticide stress in the first and second generation while transgenerational effects of maternal insecticide stress were evaluated in the second-generation beetles. Furthermore, we investigated whether transgenerational insecticide stress exposure influenced the offspring´s tolerance to the same stressor. The investigation of transgenerational effects is relevant for invasive pest insects, including the Colorado potato beetle, because they can have multiple generations per year that are exposed to the same insecticide. Based on hormesis (insecticides are known to have hormetic effects) and our previous findings^16^, exposure to insecticide stress could induce positive transgenerational effects. Offspring whose mothers were exposed to insecticide stress should be heavier and accumulate more lipids than offspring descending from mothers not exposed to the stress. Possible effects on body mass are important because higher body mass is associated with higher reproductive performance, survival and overall increased fitness (including overwintering survival)^31^. All these traits are relevant as they can facilitate the invasion of the beetle towards northern latitudes as well as generally increase its severity as a pest.

## Results

### Within-generational insecticide effects on survival in the first generation

**Figure 1:**
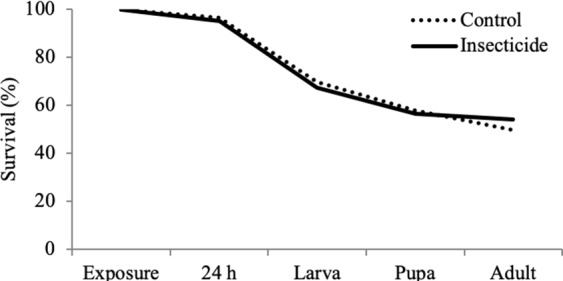
Survival (%) of insecticide stress exposed (solid line) and control (dashed line) of the Colorado potato beetles at different life stages. Insecticide stress exposure increases survival in the adult stage (0–10 days) when compared to control group.

In the first generation, after being exposed to insecticide stress for 24 hours, larval survival was 95% and 97% in the insecticide and control groups, respectively (β_21_; Fig. 1; Table 1). This high survival confirms that the insecticide exposure was sublethal. Total larval survival (i.e. survival after being exposed to insecticide for 24 h until pupation) and pupal survival were similar between the insecticide exposed and control individuals (β_22_; β_23_). However, insecticide exposed individuals were more likely to survive as adults (from adult emergence to 10 days) when compared to the control group (β_24_; Fig. 1). The odds for survival are between 0.98 and 17.99 times higher in the insecticide exposed group with 95% probability, and the probability for survival to be larger than 1 is high.

**Table 1:** Posterior means, standard deviations (i.e. SD) and 95% credible intervals for all the measured traits, and how they contribute to (a) survival in the first generation, (b) development time and body mass in the first generation, (c) survival in the second generation, (d) development time and body mass in the second generation, and (e) relative lipid content (%) in the second generation.

		Parameter	Mean	SD	2.5%	97.5%
**(a) Survival in the first generation**						
24 h larval survival	Intercept	*β*_11_	−1.06	1.972	−4.689	2.979
	Within-generational treatment: insecticide	*β*_21_	0.42	0.696	−0.896	1.807
	Larval body mass	*ε*	−0.73	0.584	−1.980	0.278
Total larval survival	Intercept	*β*_12_	−0.96	0.211	−1.382	−0.555^*^
	Within-generational treatment: insecticide	*β*_22_	0.07	0.292	−0.502	0.641
Pupal survival	Intercept	*β*_13_	−1.62	0.298	−2.231	−1.057^*^
	Within-generational treatment: insecticide	*β*_23_	−0.03	0.419	−0.861	0.791
Adult survival	Intercept	*β*_14_	−1.95	0.371	−2.722	−1.271^*^
	Within−generational treatment: insecticide	*β*_24_	−1.35	0.741	−2.890	0.024^†^
**(b) Development time and body mass in the first generation**						
Development time (log days)	Intercept	*α*_11_	3.38	0.006	3.369	3.393^*^
	Within-generational treatment: insecticide	*α*_21_	−0.002	0.007	−0.015	0.012
	Sex: male	*α*_31_	−0.02	0.007	−0.032	−0.005^*^
Emergence body mass (mg)	Intercept	*α*_12_	122.77	1.906	119.075	126.568^*^
	Within-generational treatment: insecticide	*α*_22_	−0.39	2.205	−4.660	3.953^*^
	Sex: male	*α*_32_	−18.38	2.258	−22.888	−13.963^*^
Body mass at the age of 10 days (mg)	Intercept	*α*_13_	164.93	2.900	159.289	170.493^*^
	Within-generational treatment: insecticide	*α*_23_	7.00	3.388	0.364	13.690^*^
	Sex: male	*α*_33_	−31.83	3.401	−38.483	−25.129^*^
**(c) Survival in the second generation**						
24 h larval survival	Intercept	*δ*_11_	−6.66	1.762	−10.203	−3.222^*^
	Within-generational treatment: insecticide	*δ*_21_	2.06	0.874	0.582	4.011^*^
	Transgenerational treatment: insecticide	*δ*_31_	1.02	0.947	−0.680	3.057
	Maternal 10-day mass	*δ*_41_	0.01	0.009	−0.007	0.027
	Within-generational treatment * transgenerational treatment	*δ*_51_	−1.70	1.073	−3.954	0.291
Total larval survival	Intercept	*δ*_12_	0.51	0.596	−0.670	1.689
	Within-generational treatment: insecticide	*δ*_22_	0.29	0.199	−0.096	0.682
	Transgenerational treatment: insecticide	*δ*_32_	−0.56	0.210	−0.975	−0.152^*^
	Maternal 10-day mass	*δ*_42_	−0.004	0.003	−0.010	0.003
	Within-generational treatment * transgenerational treatment	*δ*_52_	−0.32	0.293	−0.898	0.259
Pupal survival	Intercept	*δ*_13_	0.52	0.589	−0.641	1.668
	Within-generational treatment: insecticide	*δ*_23_	0.18	0.196	−0.201	0.565
	Transgenerational treatment: insecticide	*δ*_33_	−0.58	0.207	−0.986	−0.175^*^
	Maternal 10-day mass	*δ*_43_	−0.004	0.003	−0.010	0.003
	Within-generational treatment * transgenerational treatment	*δ*_53_	−0.22	0.290	−0.799	0.336
Adult survival	Intercept adult mortality (no interaction, too many had died not enough combinations to estimate the interaction)	*δ*_14_	−7.26	4.126	−15.692	0.522
	Within-generational treatment	*δ*_24_	1.00	0.971	−0.774	3.083
	Transgenerational treatment	*δ*_34_	−1.48	1.043	−3.792	0.398^†^
	Maternal 10-day mass	*δ*_44_	0.02	0.023	−0.027	0.061
**(d) Development time and body mass in the second generation**						
Development time (log days)	Intercept	*γ*_11_	3.37	0.025	3.317	3.413^*^
	Within-generational treatment: insecticide	*γ*_21_	<0.001	0.009	−0.019	0.018
	Sex: male	*γ*_31_	−0.03	0.009	−0.045	−0.008^*^
	Sex * transgenerational treatment	*γ*_41_	0.04	0.013	0.012	0.061^*^
	Transgenerational treatment	*γ*_51_	−0.02	0.011	−0.042	<0.001
	Within-generational treatment * transgenerational treatment	*γ*_61_	−0.005	0.013	−0.030	0.019
	Maternal 10-day mass	*γ*_71_	<0.001	<0.001	<0.001	<0.001
Emergence body mass (mg)	Intercept	*γ*_12_	107.83	4.973	98.018	117.455^*^
	Within-generational treatment: insecticide	*γ*_22_	1.35	1.922	−2.373	5.135
	Sex: male	*γ*_32_	−12.16	1.907	−15.885	−8.429^*^
	Sex * Transgenerational treatment	*γ*_42_	−2.76	2.512	−7.738	2.145
	Transgenerational treatment: insecticide	*γ*_52_	4.85	2.167	0.625	9.109^*^
	Within-generational treatment * transgenerational treatment	*γ*_62_	−1.99	2.525	−6.845	2.983
	Maternal 10-day mass	*γ*_72_	0.03	0.027	−0.021	0.084
Body mass at the age of 7 days (mg)	Intercept	*γ*_13_	176.37	8.760	159.017	193.616^*^
	Within-generational treatment: insecticide	*γ*_23_	3.02	3.355	−3.543	9.624
	Sex: male	*γ*_33_	−28.31	3.351	−34.988	−21.657^*^
	Sex * transgenerational treatment	*γ*_43_	−7.98	4.448	−16.644	0.829^†^
	Transgenerational treatment: insecticide	*γ*_53_	7.36	3.763	−0.002	14.525^†^
	Within-generational treatment * transgenerational treatment	*γ*_63_	−1.10	4.434	9.815	7.519^*^
	Maternal 10-day mass	*γ*_73_	0.01	0.047	−0.083	0.102
Body mass at the age of 14 days (mg)	Intercept	*γ*_14_	142.12	6.435	129.316	154.637^*^
	Within-generational treatment: insecticide	*γ*_24_	−0.37	2.471	−5.260	4.602
	Sex: male	*γ*_34_	−15.55	2.478	−20.440	−10.663^*^
	Sex * transgenerational treatment	*γ*_44_	−3.21	3.301	−9.737	3.357
	Transgenerational treatment: insecticide	*γ*_54_	4.06	2.806	−1.499	9.458
	Within-generational treatment * transgenerational treatment	*γ*_64_	0.91	3.258	−5.293	7.389
	Maternal 10-day mass	*γ*_74_	0.03	0.035	−0.042	0.094
**(e) Relative lipid content (%) in the second generation**						
Relative lipid content (%)	Intercept	*γ*_15_	−1.42	0.119	−1.650	−1.187^*^
	Within-generational treatment: insecticide	*γ*_25_	−0.03	0.046	−0.118	0.062
	Sex: male	*γ*_35_	0.06	0.030	0.004	0.122^*^
	Transgenerational treatment: insecticide	*γ*_45_	−0.04	0.042	−0.118	0.047
	Within-generational treatment * transgenerational treatment	*γ*_55_	0.06	0.060	−0.054	0.181
	Maternal 10-day mass	*γ*_65_	<0.001	0.001	−0.001	0.002
Water content (%)	Intercept	*γ*_16_	0.07	0.073	−0.070	0.213
	Within-generational treatment: insecticide	*γ*_26_	0.006	0.028	−0.050	0.062
	Sex: male	*γ*_36_	−0.02	0.019	−0.057	0.016
	Transgenerational treatment: insecticide	*γ*_46_	0.003	0.026	−0.049	0.054
	Within-generational treatment * transgenerational treatment	*γ*_56_	−0.02	0.038	−0.090	0.058
	Maternal 10-day mass	*γ*_66_	<0.001	<0.001	−0.001	<0.001
Dry mass (%)	Intercept	*γ*_17_	−0.89	0.038	−0.963	−0.815^*^
	Within-generational treatment: insecticide	*γ*_27_	0.01	0.014	−0.017	0.040
	Sex: male	*γ*_37_	−0.02	0.009	−0.041	−0.004^*^
	Transgenerational treatment: insecticide	*γ*_47_	0.005	0.013	−0.021	0.030
	Within-generational treatment * transgenerational treatment	*γ*_57_	−0.01	0.019	−0.047	0.028
	Maternal 10-day mass	*γ*_67_	<0.001	<0.001	<0.001	<0.001

Parameters with posterior probabilities greater than 95% are marked with *, and those with moderate effects are marked with^†^.

Paternal effects, as measured in a subset of the males of the study, did not affect offspring survival and had a very minor effect on offspring body mass (Supplementary Table 1). Therefore, we focus on maternal effects and pesticide treatments as the major sources of variation in the study.

### Within-generational insecticide effect on development time and adult body mass in the first generation

**Figure 2:**
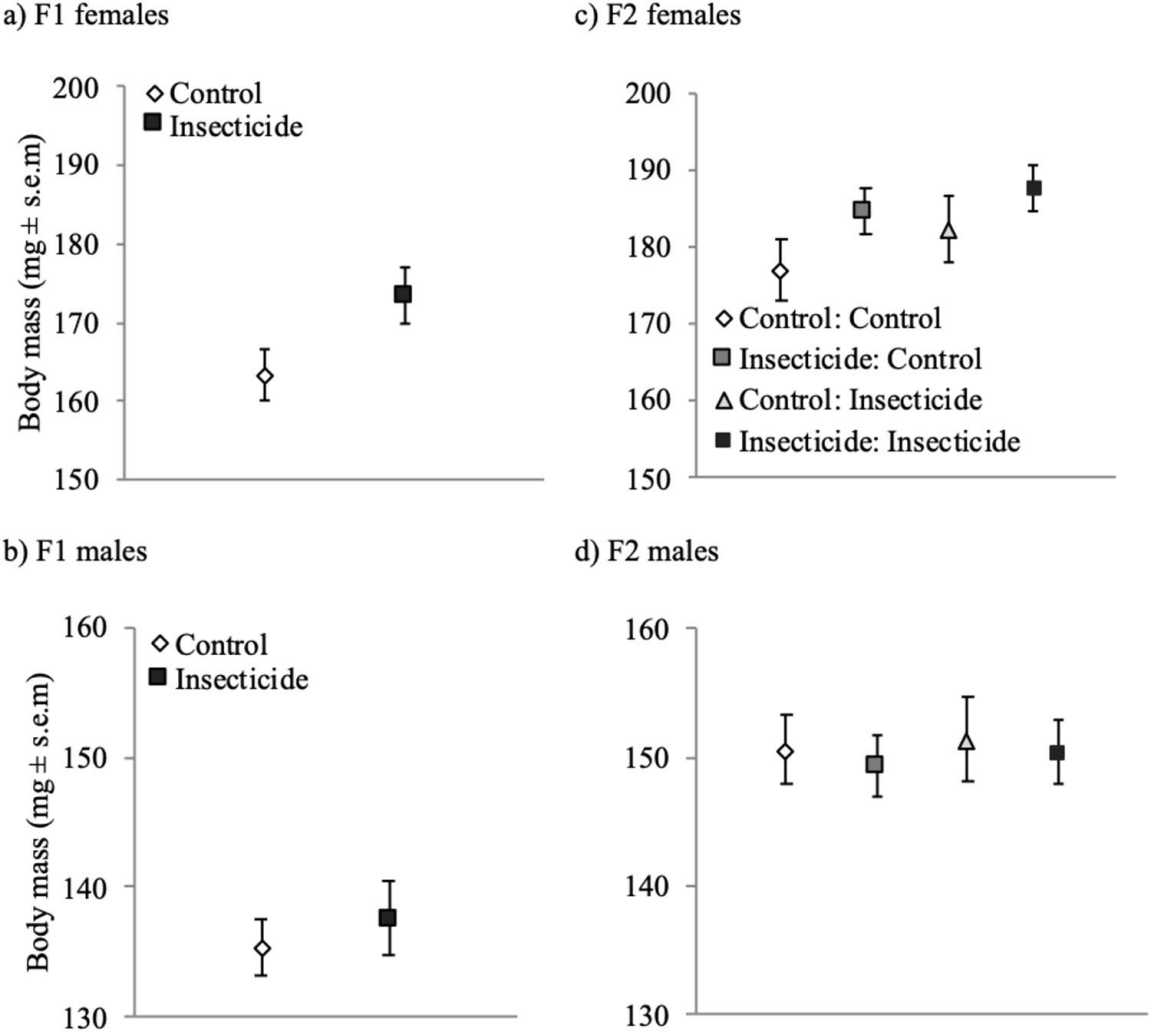
Body mass for (**a**) female and (**b**) male Colorado potato beetles in the first generation measured at the age of 10 days. Body mass for (**c**) female and (**d**) male beetles in the second generation measured at the age of 7 days. Control: Control- offspring control: maternal control (within-generational treatment: transgenerational treatment).

Development time (i.e. time in days from egg hatching to adult emergence and it is log-transformed in the model; Supp. Fig. 1a,b) was similar between insecticide exposed and control individuals (α_21_). Males developed around half a day faster than females (α_31_; Supp. Fig. 1a,b). Adult emergence body mass was similar between insecticide exposed and control individuals (α_22_; Supp. Fig. 2a,b). However, within-generational insecticide exposure had a positive effect on 10-day body mass (α_23_; Fig. 2a,b). Insecticide exposed females and males were on average 10 and 2 mg heavier than control females and males (Fig. 2a,b). Females were on average 18.4 mg heavier than males at emergence (i.e. 0-day) (α_32_; Supp. Fig. 2a,b) and on average 31.8 mg heavier at 10 days (α_33_; Fig. 2a,b).

### Within-and transgenerational insecticide effects on survival in the second generation

**Figure 3:**
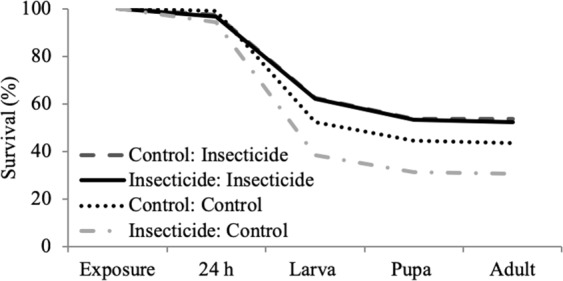
Within- and transgenerational insecticide stress effects on survival (%) between different life stages in the second generation Colorado potato beetles. Within-generational insecticide stress exposure decreases larval  and pupal survival within the 24 h when compared to control group. Transgenerational insecticide treatment decreases larval mortality when compared to larvae descending from control mothers. Insecticide: Control within-generational treatment: transgenerational treatment means that within generational treatment was insecticide stress and transgenerational treatment was control.

After the short term exposure, the 24 h larval survival was lower in the insecticide exposure group than in the control group (δ_21_; Fig. 3) but was not strongly associated with descendants from the previous generations´ (i.e. maternal) insecticide exposure (δ_31_). 24 h larval survival in the second generation after 24 h insecticide exposure was on average 0.13 times lower when compared to the control group (δ_21_; Fig. 3). There could also be a small positive interaction effect between the within- and transgenerational insecticide exposure on the 24 h larval survival (δ_51_). In other words when exposed to insecticide, the offspring of insecticide treated mothers had better survival than the offspring of control mothers.

Total larval survival was higher for larvae that descended from the insecticide exposed mothers than for those descending from the control mothers (δ_32_). The within-generational insecticide seems to slightly reduce survival of the offspring of control mothers (δ_22_) but not the survival of the offspring of the insecticide treated mothers (δ_52_). Total larval survival was on average 1.75 times higher for beetles descending from the insecticide-exposed mothers when compared to those produced by the control mothers (Fig. 3).

Survival in the pupal stage was not affected by the within- (δ_23_) or by the within- and transgenerational insecticide exposure interaction (δ_53_). However, transgenerational insecticide exposure (δ_33_) increased pupal survival on average 1.79 times (δ_33_) when compared to those in the control group. Adult survival between 0 and 14 days was not so clearly affected by the within- (δ_24_) or transgenerational insecticide exposure (δ_34_).

### Within- and transgenerational insecticide effects on the development time and body mass in the second generation

Development time (i.e. days, from egg date until adult emergence; log-transformed in the model) in the second generation was not affected by the within- (γ_21_) or transgenerational insecticide exposure (γ_51_). Males developed faster than females (γ_31_; Supp. Fig. 3a,b). There was no interaction effect on development time between the within- and transgenerational insecticide exposure (γ_61_). Within- generational insecticide exposure had no clear effect on the emergence body mass (γ_22_) or on the body mass at age of 7 (γ_23_) or 14 days (γ_24_). Transgenerational insecticide exposure, however, had a positive effect on emergence body mass (γ_52_), and on body mass at age of 7 (γ_53_; Fig. 2c,d) and 14 days (γ_54_; Supp. Fig. 4a–d). At the age of 14 days, the transgenerational insecticide exposure had a positive effect with a large variance. No clear interaction effects were observed between the within- and transgenerational insecticide exposure on the emergence body mass (γ_62_) or on the body mass at age of 7 (γ_63_) or 14 days (γ_64_). There was an indication of a sex and transgenerational treatment interaction effect on the body mass at the age of 7 days (γ_43_), suggesting that the increase in body mass is larger in females. Females were heavier than males on the emergence day (γ_32_) at the age of 7 (γ_33_; Fig. 2c,d) and 14 days (γ_34_; Supp. Fig. 4a–d).

### Within- and transgenerational effects on relative lipid content (%), water content (%), and dry mass (%) in the second generation

Relative lipid content did not differ between within- (γ_25_) or transgenerational insecticide exposure and control groups (γ_45_). No within- and transgenerational treatment interaction was found either (γ_55_). However, relative lipid content was higher for males than for females (γ_35_; Supp. Fig. 5a,b).

Water content did not differ between within- (γ_26_) or transgenerational insecticide exposure and control groups (γ_46_). No within- and transgenerational treatment interaction was found either (γ_56_). Water content did not differ between males and females (γ_36_).

Dry mass did not differ between within- (γ_27_) or transgenerational insecticide exposure and control groups (γ_47_). No within- and transgenerational treatment interaction was found either (γ_57_). Dry mass did not differ between males and females (γ_37_).

## Discussion

Invasive pest species are often repeatedly controlled by pesticides. Whereas exposure to high pesticide doses are in general lethal and form a strong selection pressure, exposure to mild, sublethal doses may lead to within- and transgenerational stress effects on survival and fitness-related traits. These effects, in turn, may contribute to the persistence of populations under stressful environments^3,32^. More importantly, higher stress tolerance or organismal flexibility of invasive species could facilitate invasions and contribute to population dynamics^3^. Here we show that exposure to sublethal pyrethroid insecticide stress can induce both positive within- and transgenerational effects manifested as higher survival and higher adult body mass of the Colorado potato beetle, which may have implications for the invasion success of the species.

Our results show that within-generational exposure to sublethal insecticide stress as larvae resulted in higher adult survival in the first generation beetles (Fig. 2). The higher adult survival of stress-exposed beetles could derive from hormetic effects. These hormetic effects might derive either from direct stimulatory responses^14,33,34^ or as an initial disruption of homeostasis, which is followed by an over-compensation response^35^. Here, the latter response pattern is more likely since the positive effect on survival was detectable in the adult stage. This indicates that exposure to stress during early stages of development can have long-lasting hormetic effects and may even increase stress resistance by contributing to survival in the adult stage. Similarly, several other studies have suggested that high stress resistance can be associated with increased longevity or survival^36,37^. Overall, high adult survival may contribute to invasion success because, in the field conditions, adults have been shown to engage in long-distance seasonal migration, which is followed by reproduction^38^, and thus insecticide-stressed individuals might be more successful. Therefore sub-lethal insecticide exposure, being a stressor, could promote invasiveness if invasive populations originate from high-stress-environments. This has been previously shown in the fresh water copepod (*Eurytemora affinis*), where invasive populations originate from more stressful environments^39^ and thus insecticide exposure could stimulate similar stress effects.

We found that stress-exposed beetles had higher 10-day body mass than control individuals in the first generation. Higher body mass could be a result of an organism trying to cope with energetic losses that derive from the insecticide detoxification which are forced to upregulate the feeding or assimilation of food^40,41^. In general, higher body mass is related to higher fitness in insects^31^. For instance, a high female body mass is often correlated with high mating success, reproduction probability, fecundity, and offspring quality as well as with high overwintering survival^42–45^. We did not explicitly measure egg laying success and thus exposure to insecticide stress could have negative effect on reproduction. However, our data shows that exposure to insecticide stress leads to higher survival and female body mass, and thus could also lead to higher reproduction rate. For example, exposure to sublethal doses of pyrethroid insecticide increased the net reproductive rate in the maize weevil (*Sitophilus zeamais*)^15^. Furthermore, if individuals from the first generation engage in the long-distance seasonal migration which typically is followed by reproduction in the Colorado potato beetle^38^. The increase in their body mass due to stress can further increase the invasion success as larger individuals could be more fertile and successful at dispersing. For example, in butterflies, body size is related to dispersal ability^46^. Overall, as both survival and body mass are very relevant fitness-related traits the observed positive within-generational stress effects on adult survival and body mass may facilitate the invasion of the beetle into novel or stressful environments.

Females in the second generation were more sensitive to stress exposure than males. This was manifested as a higher adult body mass in the females descending from insecticide-stressed mothers compared to the females descending from control mothers, whereas smaller differences in body mass were observed among males (Fig. 2b,d). Sex-specific stress effects have been shown by other studies, and they often suggest that females are more sensitive to stress than males^16,35,47,48^. Sex-specific differences in sensitivity may be due to sexual size dimorphism^49^, sex-linked pyrethroid resistance mechanism^50^ or hormetic effects that are induced by partly different mechanisms in males and females^48^.

We expected insecticide stress to have positive transgenerational effects and indeed, we found that maternal insecticide stress exposure resulted in around 17% higher larval survival (Fig. 3) and in higher female adult body mass (Fig. 2c). Positive transgenerational (hormetic) effects can be mediated via epigenetic effects. Epigenetic modifications can lead to changes in the DNA methylation patterns, can suppress or increase gene expression levels and thus affect the resistance levels to insecticides^51^. For example, Kishimoto *et al*.^52^ has shown that parental hormetic responses are transmitted to their offspring via epigenetic memory that is maintained through histone modifications. A future study could perform a genome-wide methylation profiling of possible DNA methylation polymorphisms between insecticide exposed group and control group^53^. Adaptive maternal effects are important in evolutionary dynamics because they may facilitate immediate phenotypic plasticity and/or impact both the direction as well as the rate of genetic change in response to the selection, and therefore may generate rapid phenotypic change within a population^32^. We show also that the maternal effect on the survival of their offspring is present in the larval and pupal stage and possibly also in the adult stage. Also, the higher body mass is visible already at the emergence day, which reflects the body mass of the larval period. Since we did not measure the body mass during the larval period, we can only speculate that the higher survival during the larval period results from the higher larval body mass. However, higher body mass during the larval stage can be especially important when managing invasive pest species, as insecticide applications commonly target the larval stage to minimize the crop losses.

We observed a small positive interaction effect between the within- and transgenerational insecticide exposure on larval survival after 24 hours. Although the variance of the estimated interaction effect was high, this suggests that the insecticide exposed mothers produced offspring with higher stress tolerance as their offspring survived insecticide exposure better than the offspring from the control mothers. Previously Uller *et al*.^54^ have found only weak evidence for anticipatory parental effects, particularly when the maternal environment is poor, and suggested that it might be quite rare in natural systems. However, it is also possible that our result is due to the selection for higher resistance to pyrethroids, although we used only a sublethal dosage.

Contrary to our expectation, within- or transgenerational insecticide stress did not result in higher lipid content of adult beetles. Even though the Folch method is a commonly used protocol to estimate relative lipid content, it can overestimate the lipid content^55^. Thus, small stress-induced differences in the relative lipid content might become less visible. It is also possible that there are no differences in the relative lipid content but that there could be differences in qualitative lipid composition (e.g. due to differences in lipid classes) or fatty acid profiles. Since it is known that stressed individuals can have elevated metabolic rates, and in turn increase their energy demand^56^, this might lead to differences in lipid composition.

## Conclusions

This study shows that even minute sublethal insecticide stress exposure can induce both within- and transgenerational positive effects. Thus, sublethal insecticide stress exposure can have long lasting non-desired adaptive effects, as this could lead to higher adult survival and higher body mass compared to non-exposed individuals. However, these exposed individuals will then produce offspring with higher larval and pupal survival and even higher adult body mass. Both higher larval survival and higher body mass may increase invasion potential and exacerbate management problems. It is therefore important to take into account potential performance-enhancing sub-lethal insecticide stress effects when developing pest management strategies.

## Material and Methods

### Study animal and insecticide exposure

The first generation Colorado potato beetles used in the study were the fourth generation descendants of beetles collected in potato fields in Vermont (USA; 44° 43′ N, 73° 20′ E) in 2010. Field collected beetles were mated in the laboratory and the beetles of the next generation were overwintered individually in plastic jars (100 ml, containing 60 ml of peat) in controlled climate cabinets (Type B3100; WeissTechnic, Reiskirchen-Lindenstruth, Germany) at 5 °C. Third generation adults were mated and reared at 23 °C under a long day regime of 18 h light (16 h light with 2 h of dim light to imitate sunrise and sunset, 6 h dark) to induce reproduction^57^. Beetles were fed ad libitum with fresh leaves and stems of potato (van Gogh variety). Oviposition was monitored, eggs collected and hatching checked daily.

When larvae reached the second instar (n = 245), they were randomly divided into control and insecticide treatments. Larvae were weighed (AM 100, Mettler, Columbus, OH, USA) before the treatment application. Larvae were moved onto a Petri dish (9 cm in diameter) containing a filter paper, and 1 ml of 1.59 mg/l deltamethrin solution (Trademark Decis, Aventis CropScience, Copenhagen, Denmark) was pipetted on the filter paper. The insecticide dose was chosen based on preliminary bioassays that showed around 10% mortality at the applied dose (Margus A, unpublished). In the control group, 1 ml of water was pipetted on the filter paper. After two hours a potato leaflet was supplied. Larvae were exposed to the respective treatments for 24 hours after which alive larvae were transferred onto new Petri dishes, reared individually and fed ad libitum with fresh potato leaves until pupation. Mortality was checked and recorded daily. Survival across the whole larval development is named total larval survival. Last instar larvae were placed individually in soil jars filled with peat for pupation. Adults were weighed (n = 140; ±0.1 mg; AM100, Mettler, Columbus, OH, USA) on the day of emergence and again when 10 days old. Mortality at different life stages (larva, pupa, adult) was recorded daily. Development time in days from egg hatching to adult emergence was counted.

To investigate transgenerational (i.e. maternal exposure to insecticide) stress effects on offspring performance and whether their tolerance to insecticide stress is influenced by transgenerational stress, each control male (n = 14) from the first generation was mated with 4 unrelated females, two of which were control females and the other two were insecticide-treated as larvae. So, in total we had 56 families. Each male was swapped among females every second day so that each male was mated with each of the four females at least three times. Rearing and insecticide treatment of larvae (second generation, n = 842) were conducted as described above and larvae were randomly divided into control and insecticide treatments. After emergence, second generation adults were sexed, weighed (at days 0, 7 and 14) and thereafter reared for 14 days at a constant temperature of 23 °C under a short day of 12 h light (10 h light with 2 h of dim light, 12 h dark). Mortality at different stages (larva, pupa, and adult) was recorded daily. Egg-to-adult development time was counted as above. After 14 days beetles were snap-frozen in liquid nitrogen and stored at −80 °C until lipid content analysis.

### Relative lipid content measurement

Total lipid content of the second generation adult beetles (n = 391) was measured from 14-day old adult beetles to investigate whether the within- and/or transgenerational insecticide stress exposure affects the size of energy reserves. At that age, beetles are ready to enter diapause^58,59^. The majority of lipids are located in the fat body, which is an important tissue involved in many metabolic functions and is the major energy storage in insects^60^. Total lipid content was estimated by using a modified Folch method^57,61^. Beetles were first weighed (fresh weight), then dried for 72 h at 55 °C and reweighed (dry weight). Lipids were extracted by placing beetles into small glass vials (20 ml) filled with 10 ml of chloroform: methanol solution (2:1) for 72 h at 20 °C and afterward dried for another 72 h at 55 °C. Thereafter beetles were again weighed (lean weight). Relative lipid content (%) is calculated by subtracting lean weight from dry weight and dividing by fresh weight. Water content (%) is calculated by subtracting fresh weight from lean weight and dividing by fresh weight. Dry mass (%) is calculated by dividing dry weight by fresh weight.

### Statistical analysis

**Figure 4:**
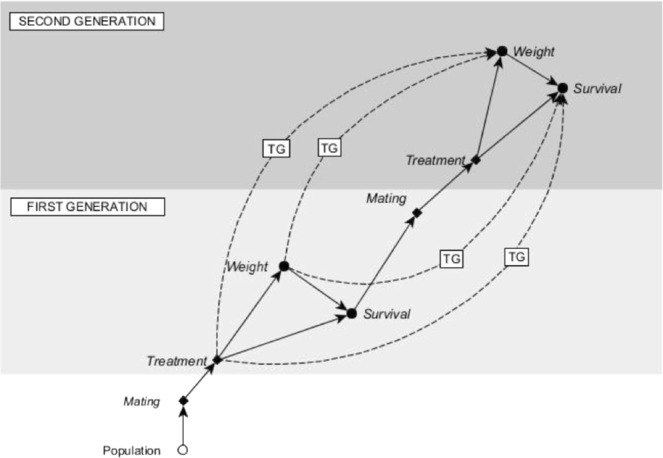
Experimental design to test the within- and transgenerational effects of sublethal insecticide exposure on survival and body mass in the Colorado potato beetle. Here the progress of the study is visualized by the ordering of the nodes. The vertical axis describes the observational time and different generations and the horizontal axis describes the causal order of events. Here the dashed arrows correspond to transgenerational (TG) causal relationships. For example, the treatment of the first generation parents has an effect on body mass of the offspring in the second generation. Open circles denote unobserved variables. Filled circles denote variables that have been measured from the sample. Similarly, diamonds denote variables that have been determined by the researcher, such as the assigned treatments or mating of each generation in this case. Our graphical presentation is a simplified version of^66^.

A Bayesian approach was chosen for the modelling task because it allows the posterior distribution of the first generation to be used as a prior for the second generation. A single Bayesian model was constructed for the whole experiment. This enables the flow of information between the generations for missing data imputation. The design of the experiment together with the associated causal assumptions and missing data mechanism are depicted in Fig. 4. Bayesian inference begins with the assumption that all of the model parameters are random variables and thus the task is to estimate their posterior distribution given some prior information about the parameters and additional evidence in the form of collected data^62^.

The effect of insecticide stress exposure in both generations and all development stages was investigated with a logistic regression model, where the survival (alive, dead) in different life stages was the response variable and within-generational treatment and transgenerational (i.e. maternal insecticide exposure) treatment were regarded as explanatory variables. Survival was analysed at each developmental stage separately because the life-cycle is partitioned into distinct stages (e.g. larva, pupa and adult). Survival probability in later life stages was modelled as conditional on having survived through the previous life stages. Thus, individuals that had died in previous stages were excluded from models for later stages in both generations. Development time (i.e. from egg hatching and hatching date to adult emergence day, in days), body mass (mg), relative lipid content (%), water content (%), and dry mass (%) were analysed with a linear model, where transgenerational treatment, within-generational treatment, and sex were considered as explanatory factors. Development time was log-transformed to better approximate it with a normal distribution. Relative lipid content (%), water content (%), and dry mass (%) were analysed with a beta model, with a logistic link for the expectation. First, a full model was fitted with parameters corresponding to interactions. If the posterior 95% credible intervals contained 0, the interactions were excluded from the final models with the exception of treatment interactions, which were kept in the model as they are one of the primary interests. Credible interaval is a interval of the shortest interval of the posterior density that contains 95% of the probability mass. In the next section, the reported intervals explicitly refer to the corresponding posterior distribution of the model parameter associated with the explanatory variable in question. The parametric forms of the final model equations are given in Supplementary Material 1.

The analysis was carried out using R^63^ and JAGS^64^ with the addition of the R-package rjags^65^. A posterior sample of size 10000 was drawn from single Markov chain with a burn-in period of two million iterations. The chain ran for an additional 5 million iterations and every 500th draw was accepted into the final sample. 10 data samples were generated using the posterior distribution to compare against the real data.

The validity of the model was checked by comparing posterior predictive distributions against the real data. The Markov chain by studying the trace plots was diagnosed and autocorrelations in addition to the 1 and 2-dimensional marginal distributions of the posterior predictive comparisons and Markov Chain Monte Carlo diagnostics are depicted in Supplementary Table 2. The estimated parameters for the statistical model are presented in Supplementary Table 3.

## Supplementary information

Margus et al Supplementary materials.
