# Supplementary material for: Sublethal Pyrethroid Insecticide Exposure Carries Positive Fitness Effects Over Generations in a Pest Insect
Source: Sci Rep. 2019 Aug 5;9:11320. doi: 10.1038/s41598-019-47473-1 (PMC6683203; doi:10.1038/s41598-019-47473-1)
Supplement: Supplementary file 1 — Margus et al Supplementary materials. [file 41598_2019_47473_MOESM1_ESM.docx]

**SUBLETHAL PYRETHROID INSECTICIDE EXPOSURE CARRIES POSITIVE FITNESS EFFECTS OVER GENERATIONS IN A PEST INSECT**

Running head: POSITIVE TRANSGENERATIONAL EFFECTS OF INSECTICIDES

Aigi Margus^1^, Saija Piiroinen^1^, Philipp Lehmann^1,2^, Santtu Tikka^3^, Juha Karvanen^3^, Leena Lindström^1^

^1^ Centre of Excellence in Biological Interactions Research, Department of Biological and Environmental Science, University of Jyväskylä, PO Box 35, FI-40014, Jyväskylä, Finland

^2^ Department of Zoology, Stockholm University, Svante Arrheniusväg 18B, SE-10691, Stockholm, Sweden

^3^ Department of Mathematics and Statistics, University of Jyväskylä, PO Box 35, FI-40014, Jyväskylä, Finland

^*^Corresponding author: Aigi Margus, Centre of Excellence in Biological Interactions Research, Department of Biological and Environmental Science, University of Jyväskylä, PO Box 35, FI-40014, Jyväskylä, Finland. Email: [aigi.margus@jyu.fi](mailto:aigi.margus@jyu.fi).

SUPPLEMENTARY TABLES, FIGURES AND MATERIALS Margus et al.

**Supplementary Table 1**. Posterior means, standard deviations (i.e. SD) and 95 % credible intervals for all the measured traits, and how they contribute to a) survival in the first generation, b) development time and body mass in the first generation, c) survival in the second generation, d) development time and body mass in the second generation, and e) relative lipid content (%) in the second generation. Parameters with posterior probabilities greater than 95% are marked with *, and those with moderate effects are marked with †.

|  |  | Parameter | Mean | SD | 2.5 % | - 1. % |
| --- | --- | --- | --- | --- | --- | --- |
| 1. Survival in the first generation | | | | | | |
| 24h larval survival | Intercept | $\beta_{11}$ | -1.06 | 1.998 | -4.739 | 3.049 |
|  | Within-generational treatment : insecticide | $\beta_{21}$ | 0.40 | 0.706 | -0.983 | 1.845 |
|  | Larval body mass | $\varepsilon$ | -0.73 | 0.591 | -2.000 | 0.296 |
| Total larval survival | Intercept | $\beta_{12}$ | -0.97 | 0.208 | -1.383 | -0.563* |
|  | Within-generational treatment : insecticide | $\beta_{22}$ | 0.07 | 0.29 | -0.507 | 0.628 |
| Pupal survival | Intercept | $\beta_{13}$ | -1.62 | 0.297 | -2.227 | -1.064* |
|  | Within-generational treatment : insecticide | $\beta_{23}$ | -0.03 | 0.419 | -0.840 | 0.805 |
| Adult survival | Intercept | $\beta_{14}$ | -1.95 | 0.369 | -2.727 | -1.280* |
|  | Within-generational treatment : insecticide | $\beta_{24}$ | -1.35 | 0.74 | -2.927 | -0.023* |
| 1. Development time and body mass in the first generation | | | | | | |
| Development time  (log days) | Intercept | $\alpha_{11}$ | 3.38 | 0.006 | 3.369 | 3.392* |
|  | Within-generational treatment : insecticide | $\alpha_{21}$ | <0.001 | 0.007 | -0.015 | 0.012 |
|  | Sex : male | $\alpha_{31}$ | -0.02 | 0.007 | -0.032 | -0.005* |
| Emergence body mass (mg) | Intercept | $\alpha_{12}$ | 122.78 | 1.897 | 119.026 | 126.477* |
|  | Within-generational treatment : insecticide | $\alpha_{22}$ | -0.37 | 2.207 | -4.710 | 4.010 |
|  | Sex : male | $\alpha_{32}$ | -18.37 | 2.247 | -22.789 | -14.003* |
| Body mass at the age of 10 days (mg) | Intercept | $\alpha_{13}$ | 164.92 | 2.908 | 159.380 | 170.666* |
|  | Within-generational treatment : insecticide | $\alpha_{23}$ | 7.02 | 3.352 | 0.425 | 13.502* |
|  | Sex : male | $\alpha_{33}$ | -31.86 | 3.436 | -38.599 | -25.084* |
| 1. Survival in the second generation | | | | | | |
| 24 h larval survival | Intercept | $\delta_{11}$ | -3.99 | 3.063 | -9.939 | 2.133 |
|  | Within-generational treatment : insecticide | $\delta_{21}$ | 2.07 | 0.852 | 0.639 | 3.986* |
|  | Transgenerational treatment : insecticide | $\delta_{31}$ | 1.03 | 0.941 | -0.656 | 3.111 |
|  | Maternal 10-day mass | $\delta_{41}$ | 0.01 | 0.009 | -0.009 | 0.027 |
|  | Within-generational treatment * transgenerational treatment | $\delta_{51}$ | -1.71 | 1.057 | -3.928 | 0.244† |
|  | Paternal 10-day mass | $\delta_{61}$ | -0.02 | 0.018 | -0.056 | 0.015 |
| Total larval survival | Intercept | $\delta_{12}$ | -0.07 | 1.102 | -2.237 | 2.113 |
|  | Within-generational treatment : insecticide | $\delta_{22}$ | 0.29 | 0.199 | -0.102 | 0.679 |
|  | Transgenerational treatment : insecticide | $\delta_{32}$ | -0.56 | 0.205 | -0.970 | -0.151* |
|  | Maternal 10-day mass | $\delta_{42}$ | <0.001 | 0.003 | -0.010 | 0.003 |
|  | Within-generational treatment * transgenerational treatment | $\delta_{52}$ | -0.32 | 0.292 | -0.891 | 0.249 |
|  | Paternal 10-day mass | $\delta_{62}$ | <0.001 | 0.006 | -0.009 | 0.016 |
| Pupal survival | Intercept | $\delta_{13}$ | -0.16 | 1.104 | -2.299 | 2.007 |
|  | Within-generational treatment : insecticide | $\delta_{23}$ | 0.18 | 0.195 | -0.208 | 0.557 |
|  | Transgenerational treatment : insecticide | $\delta_{33}$ | -0.58 | 0.209 | -0.995 | -0.167* |
|  | Maternal 10-day mass | $\delta_{43}$ | <0.001 | 0.003 | -0.010 | 0.003 |
|  | Within-generational treatment * transgenerational treatment | $\delta_{53}$ | -0.23 | 0.293 | -0.798 | 0.350 |
|  | Paternal 10-day mass | $\delta_{63}$ | 0.01 | 0.006 | -0.008 | 0.017 |
| Adult survival | Intercept adult mortality (no interaction, too many had died not enough combinations to estimate the interaction) | $\delta_{34}$ | -16.25 | 7.372 | -31.174 | -2.362* |
|  | Within-generational treatment | $\delta_{44}$ | 0.94 | 0.983 | -0.900 | 3.033 |
|  | Transgenerational treatment | $\delta_{34}$ | -1.55 | 1.086 | -3.907 | 0.408† |
|  | Maternal 10-day mass | $\delta_{44}$ | 0.02 | 0.022 | -0.021 | 0.067 |
|  | Paternal 10-day mass | $\delta_{54}$ | 0.06 | 0.04 | -0.017 | 0.140 |
| 1. Development time and body mass in the second generation | | | | | | |
| Development time  (log days) | Intercept | $\gamma_{11}$ | 3.34 | 0.048 | 3.247 | 3.437 |
|  | Within-generational treatment : insecticide | $\gamma_{21}$ | <0.001 | 0.01 | -0.019 | 0.018 |
|  | Sex : male | $\gamma_{31}$ | -0.03 | 0.009 | -0.045 | -0.008* |
|  | Sex * transgenerational treatment | $\gamma_{41}$ | 0.04 | 0.013 | 0.012 | 0.062* |
|  | Transgenerational treatment | $\gamma_{51}$ | -0.02 | 0.011 | -0.043 | -0.001* |
|  | Within-generational treatment * transgenerational treatment | $\gamma_{61}$ | -0.01 | 0.013 | -0.029 | 0.020 |
|  | Maternal 10-day mass | $\gamma_{71}$ | 0 | 0 | 0 | 0 |
|  | Paternal 10-day mass | $\gamma_{81}$ | 0 | 0 | 0 | 0.001 |
| Emergence body mass (mg) | Intercept | $\gamma_{12}$ | 73.52 | 9.583 | 54.496 | 92.270* |
|  | Within-generational treatment : insecticide | $\gamma_{22}$ | 1.01 | 1.88 | -2.644 | 4.706 |
|  | Sex : male | $\gamma_{32}$ | -12.52 | 1.871 | -16.204 | -8.856* |
|  | Sex * Transgenerational treatment | $\gamma_{42}$ | -2.16 | 2.501 | -7.053 | 2.756 |
|  | Transgenerational treatment : insecticide | $\gamma_{52}$ | 3.92 | 2.135 | -0.275 | 8.151† |
|  | Within-generational treatment * transgenerational treatment | $\gamma_{62}$ | -1.35 | 2.51 | -6.341 | 3.581 |
|  | Maternal 10-day mass | $\gamma_{72}$ | 0.06 | 0.027 | 0.004 | 0.111* |
|  | Paternal 10-day mass | $\gamma_{82}$ | 0.23 | 0.054 | 0.121 | 0.332* |
| Body mass at the age of 7 days (mg) | Intercept | $\gamma_{13}$ | 125.25 | 16.623 | 91.956 | 157.543* |
|  | Within-generational treatment : insecticide | $\gamma_{23}$ | 2.61 | 3.314 | -3.662 | 9.204 |
|  | Sex : male | $\gamma_{33}$ | -28.85 | 3.297 | -35.413 | -22.442* |
|  | Sex * transgenerational treatment | $\gamma_{43}$ | -7.11 | 4.371 | -15.513 | 1.427† |
|  | Transgenerational treatment : insecticide | $\gamma_{53}$ | 5.82 | 3.748 | -1.662 | 13.044† |
|  | Within-generational treatment * transgenerational treatment | $\gamma_{63}$ | -0.26 | 4.42 | -8.953 | 8.393 |
|  | Maternal 10-day mass | $\gamma_{73}$ | 0.05 | 0.048 | -0.047 | 0.140 |
|  | Paternal 10-day mass | $\gamma_{83}$ | 0.34 | 0.094 | 0.153 | 0.527* |
| Body mass at the age of 14 days (mg) | Intercept | $\gamma_{14}$ | 103.99 | 12.281 | 79.930 | 128.196* |
|  | Within-generational treatment : insecticide | $\gamma_{24}$ | -0.63 | 2.411 | -5.355 | 4.139 |
|  | Sex : male | $\gamma_{34}$ | -15.87 | 2.416 | -20.628 | -11.087* |
|  | Sex * transgenerational treatment | $\gamma_{44}$ | -2.56 | 3.213 | -8.917 | 3.751 |
|  | Transgenerational treatment : insecticide | $\gamma_{54}$ | 3.07 | 2.753 | -2.400 | 8.482 |
|  | Within-generational treatment * transgenerational treatment | $\gamma_{64}$ | 1.40 | 3.221 | -4.916 | 7.706 |
|  | Maternal 10-day mass | $\gamma_{74}$ | 0.06 | 0.035 | -0.014 | 0.121 |
|  | Paternal 10-day mass | $\gamma_{84}$ | 0.25 | 0.07 | 0.115 | 0.389* |
| 1. Relative lipid content (%) in the second generation | | | | | | |
| Relative lipid content (%) | Intercept | $\gamma_{15}$ | -1.09 | 0.237 | -1.545 | -0.619* |
|  | Within-generational treatment : insecticide | $\gamma_{25}$ | -0.02 | 0.047 | -0.117 | 0.068 |
|  | Sex : male | $\gamma_{35}$ | 0.06 | 0.03 | 0.004 | 0.122* |
|  | Transgenerational treatment : insecticide | $\gamma_{45}$ | -0.03 | 0.042 | -0.110 | 0.055 |
|  | Within-generational treatment * transgenerational treatment | $\gamma_{55}$ | 0.06 | 0.062 | -0.063 | 0.179 |
|  | Maternal 10-day mass | $\gamma_{65}$ | 0.00 | 0.001 | -0.001 | 0.001 |
|  | Paternal 10-day mass | $\gamma_{75}$ | 0.00 | 0.001 | -0.005 | 0.000 |
| Water content (%) | Intercept | $\gamma_{16}$ | -0.09 | 0.145 | -0.374 | 0.197 |
|  | Within-generational treatment : insecticide | $\gamma_{26}$ | 0.00 | 0.029 | -0.053 | 0.061 |
|  | Sex : male | $\gamma_{36}$ | -0.02 | 0.019 | -0.057 | 0.016 |
|  | Transgenerational treatment : insecticide | $\gamma_{46}$ | 0.00 | 0.026 | -0.053 | 0.050 |
|  | Within-generational treatment * transgenerational treatment | $\gamma_{56}$ | -0.01 | 0.038 | -0.087 | 0.061 |
|  | Maternal 10-day mass | $\gamma_{66}$ | 0.00 | 0.000 | -0.001 | 0.001 |
|  | Paternal 10-day mass | $\gamma_{76}$ | 0.00 | 0,001 | -0.001 | 0.003 |
| Dry mass (%) | Intercept | $\gamma_{17}$ | -0.96 | 0,073 | -1.106 | -0.820* |
|  | Within-generational treatment : insecticide | $\gamma_{27}$ | 0.01 | 0,014 | -0.018 | 0.039 |
|  | Sex : male | $\gamma_{37}$ | -0.02 | 0,01 | -0.042 | -0.004* |
|  | Transgenerational treatment : insecticide | $\gamma_{47}$ | 0.00 | 0,013 | -0.022 | 0.030 |
|  | Within-generational treatment * transgenerational treatment | $\gamma_{57}$ | -0.01 | 0,019 | -0.045 | 0.028 |
|  | Maternal 10-day mass | $\gamma_{67}$ | 0.00 | 0.000 | 0.000 | 0.000 |
|  | Paternal 10-day mass | $\gamma_{77}$ | 0.00 | 0.000 | 0.000 | 0.001 |

**Supplementary Table 2**. Summary statistics of the data

|  | Mean | SD | Min | Max | Q_1_ | Q_3_ |
| --- | --- | --- | --- | --- | --- | --- |
| Development time (days) in first generation | 29.2 | 1.231 | 26 | 33 | 29 | 30 |
| Emergence body mass (mg) in the first generation | 114.6 | 15.805 | 80.8 | 161.6 | 102.65 | 124.73 |
| Body mass (mg) at the age of 10 days in the first generation | 155.6 | 24.901 | 100.5 | 243.6 | 137.10 | 168.60 |
| Development time (days) in the second generation | 28.2 | 1.817 | 25 | 40 | 27 | 29 |
| Emergence body mass (mg) in the second generation | 108.9 | 14.172 | 71 | 149.2 | 99.85 | 118.65 |
| Body mass (mg) at the age of 7 days in the second generation | 166.0 | 27.041 | 97.5 | 240.5 | 147.63 | 182.95 |
| Body mass (mg) at the age of 14 days in the second generation | 140.0 | 17.949 | 100.8 | 198.9 | 128.10 | 150.15 |
| Relative lipid content (%) in the second generation | 20.9 | 4.308 | 2.6 | 30.1 | 19.14 | 23.82 |
| Water content (%) in the second generation | 50.0 | 4.412 | 42.4 | 69.3 | 47.14 | 51.61 |
| Dry mass (%) in the second generation | 29.1 | 1.658 | 25.2 | 46.3 | 28.26 | 29.77 |

**Supplementary Table 3**. Estimates of the standard deviation parameters of the models

|  | Parameter | Mean | SD | 2.5 % | 97.5 % |
| --- | --- | --- | --- | --- | --- |
| Development time (days) in first generation | $\sigma_{1}$ | 0.042 | 0.003 | 0.037 | 0.047 |
| Emergence body mass (mg) in the first generation | $\sigma_{2}$ | 13.048 | 0.798 | 11.587 | 14.763 |
| Body mass (mg) at the age of 10 days in the first generation | $\sigma_{3}$ | 18.943 | 1.213 | 16.748 | 21.484 |
| Development time (days) in the second generation | $\sigma_{4}$ | 0.062 | 0.002 | 0.0574 | 0.067 |
| Emergence body mass (mg) in the second generation | $\sigma_{5}$ | 12.404 | 0.442 | 11.583 | 13.329 |
| Body mass (mg) at the age of 7 days in the second generation | $\sigma_{6}$ | 21.552 | 0.781 | 20.080 | 23.153 |
| Body mass (mg) at the age of 14 days in the second generation | $\sigma_{7}$ | 15.781 | 0.570 | 14.710 | 16.963 |

| a) | b) |
| --- | --- |

**Supplementary figure 1**. Insecticide stress (closed symbol) and control group (open symbol) effects on development time (days ± s.e.m.) of a) female and b) male Colorado potato beetles in the first generation.

| 0 days | 10 days |
| --- | --- |
| a) | b) |
| c) | d) |

**Supplementary figure 2**. Insecticide stress (closed symbol) and control group (open symbol) effects on body mass (mg ± s.e.m.) of a-b) female and c-d) male Colorado potato beetles at the age of 0 and 10 days in the first generation. Insecticide stress exposed individuals are heavier when compared to the control group.

| **a)** | **b)** |
| --- | --- |

**Supplementary figure 3**. Within- and transgenerational insecticide stress effects on development time (days ± s.e.m.) of a) female and b) male Colorado potato beetles in the second generation. Control: Control (within-generational treatment: transgenerational treatment).

| 0 days | 7 days | 14 days |
| --- | --- | --- |
| a) | b) | c) |
| d) | e) | f) |

**Supplementary figure 4**. Within- and transgenerational insecticide stress effects on mean adult body mass (mg ± s.e.m.) of female (a-c) and male (d-f) Colorado potato beetles at the age of 0, 7 and 14 days. Control: Control (within-generational treatment: transgenerational treatment). Adult beetles descending from insecticide exposed mothers have greater body mass at the age of 0, 7 and 14 days when compared to beetles descending from control mothers.

| a) | b) |
| --- | --- |

**Supplementary figure 5**. Within- and transgenerational insecticide stress effects on relative lipid content (% ± s.e.m.) of a a) female and b) male Colorado potato beetles in the second generation. Control: Control (within-generational treatment: transgenerational treatment). Insecticide stress exposure has no within- or transgenerational effects on the relative lipid content.

**Supplementary material 1**. Equations for the models in the Bayesian analysis. All parameters priors were normal distributions with mean 0 and accuracy 0.0001 with the exception of $\sigma_{k}$, which were calculated as $\sigma_{k}=1/\sqrt{\tau_{k}}$ and the prior for the $\tau_{k}$were Gamma(0.001. 0.001)-distributions (with the parametrization used by JAGS).

Variables in the first generation:

t = Treatment, $w^{l}$= Larval body mass,$w^{10}$= 10 day body mass, s = Sex

Variables in the second generation:

$t^{2}$= Treatment, $s^{2}$= Sex

The index F(j) refers to the index i in the first generation such that individual i is the mother of individual j in the second generation.

(a)

$$P\left( Died during 24h \right)=\mathrm{logit}(\beta_{11}+\beta_{21}t_{i}+\epsilon w_{i}^{l})$$

$$P\left( Died as larva|Survived 24h \right)=\mathrm{logit}(\beta_{12}+\beta_{22}t_{i})$$

$$P\left( Died as pupa|Survived as larva \right)=\mathrm{logit}(\beta_{13}+\beta_{23}t_{i})$$

$$P\left( Died as adult|Survived as pupa \right)=\mathrm{logit}(\beta_{13}+\beta_{23}t_{i})$$

(b)

Dev. time (days, log scale) $\sim N(\alpha_{11}+\alpha_{21}t_{i}+\alpha_{31}s_{i},\sigma_{1}^{2})$

Emergence body mass (mg) $\sim N(\alpha_{12}+\alpha_{22}t_{i}+\alpha_{32}s_{i}, \sigma_{2}^{2})$

10 day body mass (mg)$\sim N(\alpha_{13}+\alpha_{23}t_{i}+\alpha_{33}s_{i},\sigma_{3}^{2})$

(c)

$$P\left( Died during 24h \right)=\mathrm{logit}(\delta_{11}+\delta_{21}t_{j}^{2}+\delta_{31}t_{F\left( j \right)}+\delta_{41}w_{F\left( j \right)}^{10}+\delta_{51}t_{j}^{2}t_{F\left( j \right)})$$

$$P\left( Died as larva \right)=\mathrm{logit}(\delta_{12}+\delta_{22}t_{j}^{2}+\delta_{32}t_{F\left( j \right)}+\delta_{42}w_{F\left( j \right)}^{10}+\delta_{52}t_{j}^{2}t_{F\left( j \right)})$$

$$P\left( Died as pupa|Survived as larva \right)=\mathrm{logit}(\delta_{13}+\delta_{23}t_{j}^{2}+\delta_{33}t_{F\left( j \right)}+\delta_{43}w_{F\left( j \right)}^{10}+\delta_{53}t_{j}^{2}t_{F\left( j \right)})$$

$$P\left( Died as adult|Survived as pupa \right)=\mathrm{logit}(\delta_{14}+\delta_{24}t_{j}^{2}+\delta_{34}t_{F\left( j \right)}+\delta_{44}w_{F\left( j \right)}^{10})$$

(d)

Dev. time (days, log scale)$\sim N(\gamma_{11}+\gamma_{21}t_{j}^{2}+\gamma_{31}s_{j}^{2}+\gamma_{41}s_{j}^{2}t_{F\left( j \right)}+\gamma_{51}t_{F\left( j \right)}+\gamma_{61}t_{j}^{2}t_{F\left( j \right)}, \sigma_{4}^{2}+\gamma_{71}w_{F(j)}^{10})$

Emergence body mass (mg) $\sim N(\gamma_{12}+\gamma_{22}t_{j}^{2}+\gamma_{32}s_{j}^{2}+\gamma_{42}s_{j}^{2}t_{F\left( j \right)}+\gamma_{52}t_{F\left( j \right)}+\gamma_{62}t_{j}^{2}t_{F\left( j \right)}+\gamma_{72}w_{F(j)}^{10}, \sigma_{5}^{2})$

7 day body mass (mg) $\sim N(\gamma_{13}+\gamma_{23}t_{j}^{2}+\gamma_{33}s_{j}^{2}+\gamma_{43}s_{j}^{2}t_{F\left( j \right)}+\gamma_{53}t_{F\left( j \right)}+\gamma_{63}t_{j}^{2}t_{F\left( j \right)}+\gamma_{73}w_{F\left( j \right)}^{10},\sigma_{6}^{2})$

14 day body mass (mg) $\sim N(\gamma_{14}+\gamma_{24}t_{j}^{2}+\gamma_{34}s_{j}^{2}+\gamma_{44}s_{j}^{2}t_{F\left( j \right)}+\gamma_{54}t_{F\left( j \right)}+\gamma_{64}t_{j}^{2}t_{F\left( j \right)}+\gamma_{74}w_{F\left( j \right)}^{10},\sigma_{7}^{2})$

(e)

Relative lipid content (%) $\sim Beta(a_{Lj},b_{Lj})$, where $a_{Lj}=\mu_{Lj}\phi_{L}$, $b_{Lj}={(1-\mu}_{Lj})\phi_{L}$and

$$\mathrm{logit}\left( \mu_{Lj} \right)=\gamma_{15}+\gamma_{25}t_{j}^{2}+\gamma_{35}s_{j}^{2}+\gamma_{45}t_{F\left( j \right)}+\gamma_{55}t_{j}^{2}t_{F\left( j \right)}+\gamma_{65}w_{F\left( j \right)}^{10}$$

Water content (%) $\sim Beta(a_{Hj},b_{Hj})$, where $a_{Hj}=\mu_{Hj}\phi_{H}$, $b_{Hj}={(1-\mu}_{Hj})\phi_{H}$and

$$\mathrm{logit}\left( \mu_{Hj} \right)=\gamma_{16}+\gamma_{26}t_{j}^{2}+\gamma_{36}s_{j}^{2}+\gamma_{46}t_{F\left( j \right)}+\gamma_{56}t_{j}^{2}t_{F\left( j \right)}+\gamma_{66}w_{F\left( j \right)}^{10}$$

Dry mass (%) $\sim Beta(a_{Mj},b_{Mj})$, where $a_{Mj}=\mu_{Mj}\phi_{M}$, $b_{Mj}={(1-\mu}_{Mj})\phi_{M}$and

$$\mathrm{logit}\left( \mu_{Mj} \right)=\gamma_{17}+\gamma_{27}t_{j}^{2}+\gamma_{37}s_{j}^{2}+\gamma_{47}t_{F\left( j \right)}+\gamma_{57}t_{j}^{2}t_{F\left( j \right)}+\gamma_{67}w_{F\left( j \right)}^{10}$$
